# Modulation of TLR4 Sialylation Mediated by a Sialidase Neu1 and Impairment of Its Signaling in *Leishmania donovani* Infected Macrophages

**DOI:** 10.3389/fimmu.2019.02360

**Published:** 2019-10-09

**Authors:** Joyshree Karmakar, Saptarshi Roy, Chitra Mandal

**Affiliations:** Cancer Biology and Inflammatory Disorder Division, CSIR-Indian Institute of Chemical Biology, Kolkata, India

**Keywords:** cathepsin A, cytokines, innate immunity, lysosomal sialidase, Neu1, sialic acids, TLR4, visceral leishmaniasis

## Abstract

Altered sialylation is generally maintained by a fine balance between sialidases and sialyltransferases, which plays an essential role during disease pathogenesis. TLR4 is a membrane-bound highly sialylated glycoprotein predominantly having α2,3-linked sialic acids. It is one of the most important client molecules in the anti-leishmanial innate immune arm. Here, we initiated a comprehensive study on the modulation of TLR4 sialylation in *Leishmania donovani* (*L. d*)-infected macrophages by a mammalian sialidase/neuraminidase-1 (Neu1) having substrate specificity toward α2,3-linked sialic acids. We observed reduced membrane-associated Neu1 with its decreased enzyme activity in infected macrophages. Moreover, we demonstrated reduced association of Neu1 with TLR4 leading to enhanced sialylation of TLR4 in these infected cells. Conversely, Neu1 over expression exhibited enhanced association of TLR4 with Neu1 leading to reduced sialylation which possibly linked to increased association of TLR4 with its downstream adaptor protein, MyD88. This, in turn, activated downstream MAP kinase signaling pathway, with enhanced nuclear translocation of NFκB that resulted in increased genetic and protein levels expression of Th1 cytokines and effector molecule nitric oxide secretion which ultimately leads to reduced parasite burden in macrophages. This was further validated by Neu1 silencing in infected macrophages which reversed such a situation. Such events strongly confirm the importance of Neu1 in modulation of TLR4 sialylation during parasite infection resulting in impairment of innate immune response. Furthermore, decreased membrane-bound Neu1 in infected macrophages could be attributed to its reduced tyrosine-phosphorylation as well as diminished association with cathepsin A. Both these phenomenon possibly play significant roles in inhibiting translocation of the sialidase from cytosol to membrane. Taken together, our study first time demonstrated impaired translocation of cytosolic Neu1 to the membrane of *L. donovani*-infected macrophages due to impaired phosphorylation of this enzyme. This novel finding establishes a link between enhanced α2,3-linked sialic acids on TLR4 and reduced membrane-bound Neu1 which plays a significant role for inhibiting downstream signaling to establish successful infection in the host cells.

## Introduction

Leishmaniasis is a neglected tropical disease caused by an obligate intracellular protozoan parasite *Leishmania* (1). It manifests in three different forms namely cutaneous, mucocutaneous and visceral leishmaniasis. However, the visceral form (VL), caused by *Leishmania donovani*, is the most dangerous and fatal if left untreated. It is endemic in many parts of India. Approximately one million new cases occur every year with 20,000–30,000 deaths and among these 90% of the cases are from India and Africa (2).

Sialic acids are terminally present in the glycan chains of glycoproteins and glycolipids on the surface of various cells (3–5). Consequently sialylation level affects the activities of immune cells under different physiological and pathological conditions mainly in cancer (6–12) and parasitic diseases (13–17). Most importantly, these sialic acids are involved in the modulation of intracellular communications (18) and host-pathogen interactions in *L. donovani, Pseudomonas aeruginosa*, and *Trypanosoma cruzi* infection (19–23). A fine balance between sialylation and desialylation machinery is maintained by sialyltransferases and sialidases/neuraminidases to sustain the level of cell surface sialic acids (24–27). Sialyltransferase mediates the transfer of sialic acids to glycan chains. On the other hand, sialidases desialylate different cell surface receptors and regulate their function (28–30). Till date, four sialidases have been recognized. Lysosomal Neu1 desialylates glycoproteins with α2,3-linked sialic acids, cytosolic Neu2 catabolizes glycoconjugates with α2,6-linked sialic acids, membrane-bound Neu3 cleaves gangliosides, and luminal Neu4 catalyzes both gangliosides and glycoproteins (31). We have recently established the role of cytosolic Neu2 on the plasma membrane in pancreatic cancer (29) and membrane-bound Neu3 in leukemia (30).

During monocyte to macrophage differentiation, the expression of lysosomal Neu1 is upregulated and targeted to the plasma membrane which enhanced the phagocytic capacity of these cells to uptake bacteria suggesting its important role in immune activation (32). Additionally, LPS stimulation induces Neu1 translocation to the macrophage cell surface (33). This lysosomal Neu1 is also found on the surface of activated T cells where it influences immune functions and exhibits an immunomodulatory role (34).

Macrophages recognize between self and non-self-pathogens by expressing pattern recognition receptors (PRRs) like Toll-like receptors (TLRs) on their surfaces (35, 36). They are the sensors of the innate immune system that can recognize invading pathogens and elicit an immune response (37, 38). Only TLR2 and TLR4 are expressed on the surface of macrophages (39). Although TLRs are highly glycosylated, the presence of sialic acids has not been reported except for TLR4. This sialylated glycoprotein exhibited α2,3-linked sialic acids attached to β-galactosyl residues (40).

*Leishmania donovani* resides safely inside the macrophages, possibly by impairing the host's innate and adaptive immunity (41). *Leishmania donovani* infection is known to deactivate TLR4-mediated innate immune response (42–45). However, the role of cell surface sialic acids in dampening such immune response is still elusive. Additionally, whether the bulky terminal α2,3-linked sialyl residues on TLR4 prevent its association with other adaptor molecules thereby leading to deactivation of TLR4 signaling during this parasite infection has not been established yet. Alternatively, the interaction of *Leishmania* with TLR4 may also be hampered due to the presence of these bulky sialic acid moieties which remains to be properly investigated. No report so far exists exhibiting any correlation between the status of TLR4-sialylation and its signaling during *L. donovani* infection. Accordingly, here we addressed the role of Neu1 in immune modulation during this parasite infection.

Here, we demonstrated that sialylation is enhanced during *L. donovani* infection with decreased Neu1 on the infected macrophages. Such reduced membrane-bound Neu1 resulted in inefficient removal of sialic acids ensuing hypersialylation of TLR4 which ultimately impaired innate immune activation. This was validated by Neu1 overexpression in macrophages followed by *L. donovani* infection. These cells exhibited enhanced association of both TLR4 and Neu1 along with TLR4 and MyD88. Further study revealed that overexpressed Neu1 was able to rescue these cells from the effect of impaired TLR4 signaling as indicated by activation of downstream MAP kinase signaling pathways such as p-JNK, p-ERK, and p-P38 with enhanced nuclear translocation of NFκB that resulted in increased expression of Th1 cytokines and nitric oxide secretion leading to reduced parasite burden in these macrophages.

## Materials and Methods

### Ethics Statement

All the animal experiments were carried out in accordance with the National Regulatory Guidelines issued by Committee for the Purpose of Control and Supervision of Experiments on Animals (CPCSEA), Ministry of Environment and Forest, Government of India. Use of Syrian Golden hamsters and Balb/c mice were approved by the Institutional Animal Ethics Committee of CSIR-Indian Institute of Chemical Biology, Kolkata, India with license number 147/1999/CPCSEA. Animals were housed under the standard condition such as temperature (25 ± 1°C), relative humidity (55 ± 10%) and 12 h/12 h light/dark cycles and fed with the standard diet.

### Chemicals

Fluorescein isothiocyanate (FITC), bovine serum albumin (BSA), 4′, 6-diamidino-2-phenylindole (DAPI), Giemsa stain, and 2′-(4-Methylumbelliferyl)-α-D-N-acetylneuraminic acid (4MU-NeuAc), 4-methylumbelliferone (MU) were from Sigma (St. Louis, MO). Mounting medium was from Amersham Biosciences (Uppsala, Sweden); *Maackia amurensis* lectin II (MALII) and *Sambucu snigra* lectin (SNA) were from Vector Labs, and DyNAmo Color Flash SYBR Green qPCR kit was from Thermo Scientific (Rockford, IL). Anti-Neu1, cathepsin A was from Invitrogen (Carlsbad, CA), Anti-TLR4 antibody was from Santa Cruz Biotechnology (MTS510). Anti-Myd88 was from R&D Systems (MN, USA). Anti-phosphotyrosine antibody was from Biolegend (San Diego, CA). All the cytokine ELISA kits were from BD pharmingen, Neu1 plasmid DNA was from Origene (MR1049), Neu1 shRNA was obtained from Sigma (SHCLNG-NM010893), RNeasy Mini Kit was from Qiagen (Limburg, Netherlands); Reverse Transcriptase Kit was from Promega (WI, USA). All other antibodies were from Cell Signaling Technologies (Danvers, MA) unless indicated otherwise.

### Parasite Culture

Promastigotes of an Indian *L. donovani* (*L. d*) strain AG83 (MHOM/IN/1983/AG83) were maintained in M-199 medium containing HEPES buffer (20 mM, pH 7.5) supplemented with 10% heat-inactivated fetal calf serum (FCS, v/v), and gentamycin sulfate (200 μg/ml) at 22°C as described before. To ensure the virulence, stationary phase promastigotes (2 × 10^7^ cells/100 μl) were routinely passaged in the golden hamster. After 4–6 weeks, hamsters were sacrificed and spleen tissue was collected in sterile phosphate buffer saline (PBS) to obtain amastigotes as described earlier (46).

### Cell Culture

Murine macrophage cell line J774A.1 obtained from ATCC was cultured in Iscov's modified Dulbeccos Medium (IMDM) supplemented with 10% heat-inactivated FCS and antibiotic- antimycotic solutions (complete medium) at 37°C with 5% CO_2_. To maintain confluency, they were sub cultured every 3–4 days as described previously (47).

### Determination of Linkage-Specific Sialic Acids on Macrophages by Flow Cytometry (FACS)

Macrophages J774.A1 cells (1 × 10^6^) were infected with stationary phase promastigotes at a multiplicity of infection 1:10 (macrophage: parasite) for 4 h. To remove unbound and/or loosely adhered parasites on the membrane of infected cells, they were repeatedly washed with IMDM. The presence of parasites on the surface of the infected macrophages was monitored under the microscope after each wash. Thereafter, the infection was allowed for additional 8 h.

Cells were extensively washed and resuspended in lectin-binding buffer (20 mM Tris, 0.5 M NaCl, 2.0 mM MnCl_2_, 2.0 mM MgCl_2_, 2.0 mM CaCl_2_) (48) and incubated at 4°C separately with FITC-conjugated MALII (5.0 μg/ml) and FITC-SNA (4.0 μg/ml) which recognizes α2,3 and α2,6-linked sialic acids, respectively for 1 h (49). Thereafter, cells were washed twice with lectin binding buffer (300 μl, 2X) and finally resuspended in the same buffer. FITC positivity was acquired by FACS (FACS Calibur, BD Bioscience) and 10,000 cells were analyzed in CellQuestPro software.

Uninfected or infected J774.A1 cells were similarly processed after staining with anti-Neu1 antibody for 1 h at 4°C. This was followed with Alexa Fluor 488 conjugated secondary antibody for 1 h. Status of cell surface-bound Neu1 was demonstrated through Alexa Fluor 488-positivity acquired by FACS.

### Western Blot and Immunoprecipitation Analysis

Uninfected and infected J774.A1 (2 × 10^6^) cells were resuspended in PBS, containing pepstatin A (1.0 μg/ml), aprotinin (10 μg/ml), and 10 μg/ml leupeptin (Lysis buffer). They were lysed by sonication (Qsonica-LLC, XL-2000 series, Newtown, CT, USA) and centrifuged at 800 × g for 10 min. The supernatant obtained was centrifuged at 1, 000, 00 × g for 30 min to separate cytosolic and membrane fractions (50). Protein was estimated using Bicinchoninic acid assay using bovine serum albumin (BSA) as standard.

Membrane fractions (100 μg/lane) were separated on gradient SDS-PAGE (7–15%) and transferred onto a PVDF membrane for glycoprotein analysis. The membrane was blocked with 4% desialylated BSA in tris buffer saline (TBS) for 1 h and incubated with biotinylated MALII and SNA separately for overnight at 4°C. They were additionally blotted with biotinylated Peanut Agglutinin (PNA). These were subsequently probed with avidin-HRP and signal was detected by West-pico enhanced chemiluminescent substrate (ECL) system (Pierce, Thermo Scientific, USA) as described earlier (41).

For detection of signaling molecules, an equal amount of protein from each sample was separated in SDS-PAGE (10%), transferred as before, blocked with TBS-2%BSA and incubated with specific primary antibody of p-JNK, JNK, p-ERK, ERK, p-p38, p38 (1:1,000 dilutions) overnight at 4°C. The blots were incubated with respective species-specific HRP-conjugated secondary antibody (1:1,000 dilutions) and developed by ECL kit.

For immunoprecipitation experiments, cell lysates (300 μg/lane), cytosolic (300 μg/lane), and membrane (400 μg/lane) fractions were incubated with specific antibodies (1:100) overnight at 4°C. The antibody-bound complex was pulled down with Sepharose-4B-protein-A. This was followed by washing with PBS and the complex was resolved in SDS-PAGE (10%) under non-reducing condition followed by western blotting. The densitometry score for each band was determined by ImageJ software and plotted as band intensities.

### Measurement of Sialidase Activity

Sialidase activity of uninfected and *L. donovani.*-infected macrophages was determined using equal amount (100 μg) of protein from cytosol and membrane fractions by using a fluorimetric assay with an artificial substrate 4-MU-Neu5Ac (400 μM) as described elsewhere (29, 30). The reaction mixture was incubated at 37°C for 1 h in sodium acetate buffer (50 mM, pH 4.5). The reaction was terminated with 1.0 ml solution containing 0.133 M glycine, 0.06 M NaCl, and 0.083 M Na_2_CO_3_, pH 10.7. Liberated 4-methylumbelliferone was measured with a spectrofluorometer with excitation at 355 nm and emission at 460 nm. A standard curve of increasing amounts of 4-methylumbelliferone was used to determine the amount of 4-methylumbelliferone liberated from 4-MUNANA. Enzymatic activity was expressed as μM of product per h/mg protein.

### Confocal Microscopy

Uninfected or infected J774.A1 cells (2 × 10^4^) were adhered on coverslips for 48 h, washed with PBS and fixed with 4% paraformaldehyde for 15 min. Fixed cells were stained with either FITC-MALII or FITC-SNA. Additionally, cells were also permeabilized by 0.5% TritonX-100. Cells before and after permeabilizations were incubated with rabbit anti-Neu1 antibody followed by staining with rabbit Alexafluor 488 antibody for visualizing the membrane and cytosolic Neu1. They were mounted in mounting media containing DAPI to stain nucleus and examined on a Zeiss inverted confocal microscope (51).

### Transfection of Neu1 Plasmid and Neu1 shRNA Into Macrophages

J774.A1 (1 × 10^6^ /well) in IMDM at 80% confluence were transfected with Neu1 plasmid DNA or Neu1 shRNA by the transfection reagent, lipofectamine-ltx according to the manufacturer's instructions in a six-well-plate for 6 h. The transfection mixture was replaced with fresh medium. Mock transfection was used as the control in all the experiments. They were infected with stationary phase promastigotes for 4 h.

### Quantitation of Cytokines and Nitric Oxide (NO)

Neu1-transfected and mock-transfected J774A.1 (1 × 10^6^ /well) were infected with stationary phase promastigotes of *L. donovani* at 1:10 ratio for 4 h in a six well plate. Unbound parasites were washed out and infection was allowed for additional 20 h. Cell-free culture supernatant was collected and assessed for the accumulated cytokines using a sandwich ELISA kit following the manufacturer's protocol (41) and nitrite using Griess reaction (52, 53).

### Genetic Expression Profiling by Real Time PCR

Total RNA from Neu1-transfected and mock-transfected J774A.1 cells was extracted using the RNeasy mini kit following the manufacturer's instruction. First strand cDNA was synthesized by ImPromII-Reverse transcription system according to the manufacturer's protocol. Real-time PCR was performed with specific primers for Th1 (IL-12 and IFNγ), Th2 cytokines (IL-4, IL-10, and TGF-β), and iNOS obtained from Eurofins Genomics India Pvt. Ltd. (Table 1) using a DyNAmo Flash SYBR Green qPCR Kit. Relative amounts of target mRNA were quantitated using the Light Cycler 96 (Roche) software with 18S rRNA as an internal control. Data were expressed as a fold change compared with uninfected control using the comparative cycle threshold (CT) method (54).

**Table 1:** List of primers.

**Primer**		**Primer sequence (5′-3′)**	**Tm (°C)**
IL-12	Forward	CAGGATGGAGAATTACAGGA	51.2
	Reverse	GTTATTGAGGGCTTGTTGAG	51.2
IFNγ	Forward	CAGGTGGCATAGATGTGGAAGA	54.7
	Reverse	GTGGGTTGTTGACCTCAAACTT	55.7
iNOS	Forward	ACCTGAAAGAGGAAAAGGAC	52.2
	Reverse	GGAGCCATAATACTGGTTGA	51.7
IL-4	Forward	CAACGAAGAACACCACAGAGAG	55.6
	Reverse	GATGTGGACTTGGACTCATTCA	54.6
IL-10	Forward	CTAACGGAAACAACTCCTTG	51.0
	Reverse	GAAAGGACACCATAGCAAAG	51.2
TGF-β	Forward	CCCTAGATTTTGACTTGCAC	51.2
	Reverse	GCCCAGTCACTAAGACTCTG	54.7
18s rRNA	Forward	GCTCATTAAATCAGTTATGG	46.0
	Reverse	ACTACCATCGAAAGTTGATA	46.0

### Measurement of Intracellular Parasites in Peritoneal Macrophages by Giemsa Staining

Resident peritoneal macrophages from Balb/c mice (8–10 weeks old) were obtained by injecting 5–10 ml chilled PBS supplemented with 3% FCS into the peritoneal cavity (46). The peritoneal exudates cells were pulled out using a syringe, centrifuged (200 × g, 5 min), and washed with PBS. These cells (5 × 10^5^ cells) were grown on 22 mm glass coverslip in complete medium for 20 h. After washing non-adherent cells, adherent cells were transfected followed by infection with stationary phase promastigote. Unbound parasites were washed after 4 h and infection was allowed for further 20 h. After infection, the cells were washed with PBS, methanol fixed and stained with Giemsa. The amastigotes were counted manually under a light microscope (EVOS, Life Technologies) at oil immersion (100x) both in Neu1-transfected and mock-transfected cells (55).

### Statistical Analysis

All the data were the mean value from three independent experiments and statistical analysis was performed using Graph Pad Prism 5. Standard error bars represent the standard deviation of the mean (±S.D.) and statistically significant differences were determined using students *t*-test. *p ≤ 0.05, **p ≤ 0.01, and***p ≤ 0.001 were considered significant.

## Results

### Enhanced Cell Surface Sialylation in *Leishmania donovani* (*L. d*) Infected Macrophages

**Figure 1:**
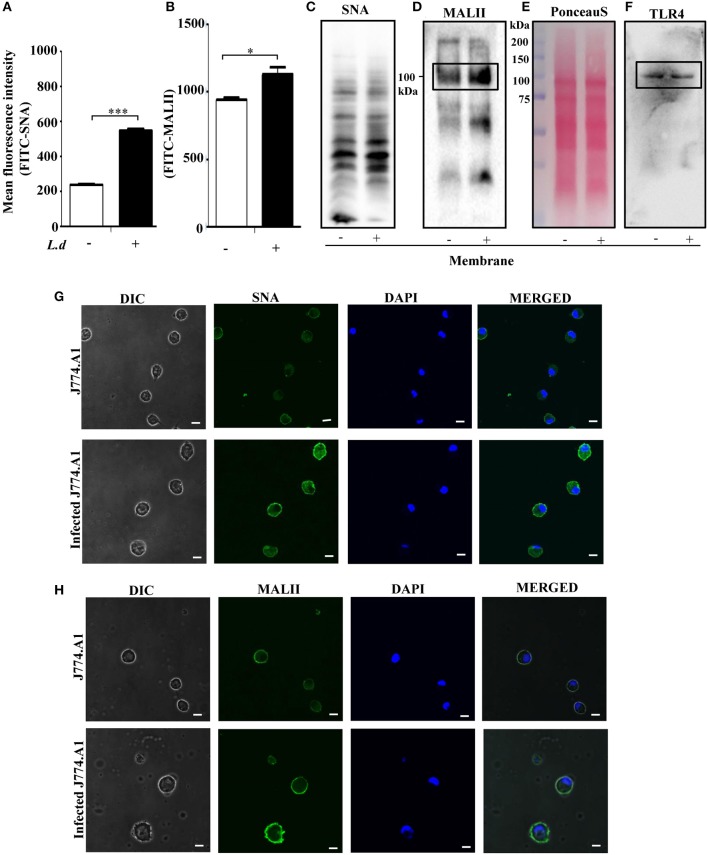
Enhanced cell surface sialylation during *Leishmania donovani* (*L. d*) infection. **(A,B)** For the analysis of linkage-specific sialic acids on macrophages during *L. donovani* infection, J774.A1 cells were either left uninfected or infected with stationary phase promastigotes in a six well-plate at 1:10 ratio for 12 h. Cells were processed for lectin-binding either with SNA-FITC **(A)** or MALII-FITC **(B)**, respectively as described in Materials and Methods. Status of sialic acids on the cell surface was demonstrated through FITC-positivity. **(C,D)** Status of sialoglycoproteins on the surface of *L. donovani*-infected macrophages were assessed by lectin-blotting of membrane fractions using biotinylated SNA **(C)** and MALII **(D)** as described in materials and methods. Ponceau S stained blot indicates equal loading **(E)**. **(F)** The blot as in **(D)** was stripped and reprobed with anti-TLR4 antibody. The protein band in the box represents the TLR4 band. **(G,H)** Adhered uninfected or infected cells were fixed, stained with FITC-SNA **(G)** and FITC-MALII **(H)**. DAPI stained the intact nucleus in blue. Sialic acids were visualized in a Zeiss inverted confocal microscope. Scale bar = 20 μM. Representative images exhibited enhanced α2,3 and α2,6-linked sialic acids on the surface of *L. donovani* infected macrophages. Each determination was expressed as mean ± SD for three independent experiments. A Student's *t*-test was used to evaluate statistical significance; *p ≤ 0.05 and ***p ≤ 0.001 when compared to uninfected cells.

Altered sialylation is closely associated with different diseases. However, its correlation with impairment of innate immunity was not adequately studied in *L. donovani* infection. Therefore, initially, we checked the status of sialylation on macrophages during infection by FACS analysis. Accordingly, murine macrophage cells (J774.A1) were infected with stationary phase promastigotes. Infected cells exhibited enhanced α2,6 and α2,3-linked sialic acids as depicted by higher binding with SNA and MALII, respectively. The mean fluorescence intensity (MFI) of SNA bound cells increased significantly (*P* ≤ 0.001) from 238.7 ± 7.055 to 534.3 ± 6.386 (Figure 1A) and that of MALII (*p* ≤ 0.05) from 961.3 ± 33.96 to 1163 ± 37.17 (Figure 1B). This was further corroborated by lectin blotting. A few SNA-bound (Figure 1C) and MALII-bound (Figure 1D) membrane sialoglycoproteins showed the enhanced expression on infected cells compared to uninfected macrophages. Ponceau S staining of the same blot was used to show equal loading (Figure 1E). One of the upregulated α2,3 linked sialoglycoprotein on the surface of *L. donovani*. infected macrophages exhibited an apparent molecular weight of ~100 kDa (Figure 1D) which matched with the size of TLR4 protein as shown by western blotting (Figure 1F). This was further confirmed by confocal microscopy. Infected macrophages showed enhanced binding to FITC-SNA (Figure 1G) and FITC-MALII (Figure 1H) as indicated by more green color on the cell surface compared to uninfected macrophages. Macrophage nucleus was stained with DAPI in blue.

### Reduced Neu1 Expression in the Membrane of *L. donovani* Infected Macrophages

**Figure 2:**
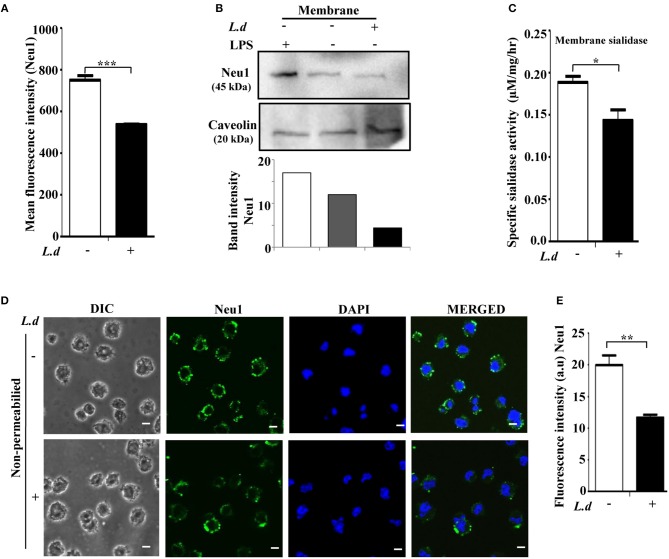
Cell surface Neu1 is reduced during *Leishmania donovani* infection. **(A)** Uninfected or infected J774.A1 cells were stained with anti-Neu1 antibody, followed by Alexa Fluor 488 conjugated secondary antibody as described in Materials and Methods. Representative bar graph indicated reduced mean fluorescence intensity (MFI) on the cell surface of infected compared to uninfected cells. **(B)** Membrane fractions of uninfected and *L. donovani* infected as well as LPS stimulated J774.A1 cells were resolved by SDS-PAGE. Status of membrane-bound Neu1 protein was analyzed by western blot using anti-Neu1 antibodies as described in Materials and Methods. Caveolin was used to check the purity of the membrane fractions as well as for loading control. **(C)** Decreased sialidase activity of membrane protein (100 μg) on the surface of *L. donovani* infected macrophages was measured at pH 4.5 using MU-Neu5Ac as substrate as described in materials and methods. **(D)** Uninfected or infected J774.A1 cells (2 × 10^4^) were fixed in paraformaldehyde and processed as mentioned in Materials and Methods. The image was visualized in an inverted confocal microscope exhibiting reduced fluorescence of Neu1 on the surface of *L. donovani* infected macrophages. Scale bar = 20 μM. The fluorescence intensity in arbitrary units (a.u) of Neu1 was determined using ImageJ software **(E)**. Data were derived from three independent experiments and presented as mean values ± SD. Significance *p ≤ 0.05, **p ≤ 0.01, and ***p ≤ 0.001.

Enhanced cell surface sialylation upon *L. donovani* infection, prompted us to monitor the status of membrane-bound Neu1 on infected J774.A1 cells by flow cytometry (Figure 2A). Neu1 exhibited a significant (*p* ≤ 0.001) lower binding on the surface of infected macrophages than in uninfected cells (MFI 536.7 ± 3.180 vs. 750.7 ± 21.46, respectively).

For further confirmation, membrane fractions were isolated and checked for level of Neu1 by western blot. Densitometry analysis showed a ~2.5-fold decrease of Neu1 in the membrane fraction of infected cells compared to uninfected cells (Figure 2B). Caveolin was used to show the purity of the plasma membrane. As expected, LPS-stimulated macrophages exhibited a higher amount of Neu1 on the membrane under similar conditions, which was used as a positive control.

In order to check whether altered Neu1 protein level is associated with a change in its enzyme activity, macrophages were infected with *L. donovani* and the lysates were used as the source of sialidase enzyme. The sialidase activities in uninfected vs. infected macrophages were determined using an exogenous substrate 4-MU-NANA. This assay showed a significant (*p* ≤ 0.05) reduction in the sialidase activity in the membrane fraction of infected macrophages (Figure 2C).

This finding was further confirmed by confocal microscopy (Figure 2D). Immunostaining with anti-Neu1 antibody followed by Alexa fluor 488 secondary antibodies exhibited significantly (*p* ≤ 0.01) decreased expression of Neu1 on the cell surface of infected macrophages as indicated by less green color when compared to uninfected macrophages. DAPI stained the intact cell nuclei. The mean intensity graph of green fluorescence was shown in Figure 2E.

### Enhanced α2,3 Sialyl Residues on TLR4, Reduced Neu1-TLR4 Association and TLR4-MyD88 Complex Recruitment in Infected Macrophages

**Figure 3:**
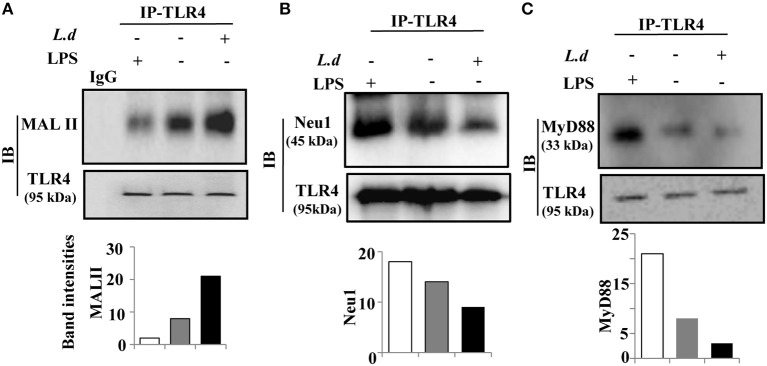
Reduced association of Neu1 leads to hypersialylation of TLR4 and decreased interaction with MyD88 during *Leishmania donovani* infection. **(A–C)** The cell lysates obtained from uninfected or infected J774.A1 cells (1 × 10^6^/well) were incubated with anti-TLR4 antibody or control isotype IgG for overnight and immunoprecipitated with protein A beads. The precipitates were analyzed by SDS-PAGE under non-reducing conditions and subsequently probed with biotinylated MALII or anti-TLR4 antibody **(A)** and processed as described in Materials and Methods. Additionally, the immunoprecipitates were processed using anti-Neu1 **(B)** and anti-MyD88 **(C)** antibodies LPS-stimulated macrophages as positive and IgG as negative controls were used. The experiments were repeated thrice and representative immunoblots are shown. The densitometric score for each band was determined by ImageJ software and plotted as band intensities. IP, immunoprecipitation; IB, immunoblot.

We had observed enhanced cell surface sialylation on infected macrophages (Figures 1A,B). As TLR4 is a membrane-bound sialylated glycoprotein, accordingly, we checked the sialylation status of TLR4 in *L. donovani*-infected macrophages. We immunoprecipitated the cell lysates from both uninfected and infected macrophages with anti-TLR4 antibody and immunoblotted with MALII. An enhanced α2,3-sialyl residues on TLR4 were observed in infected macrophages compared to uninfected cells (Figure 3A). LPS, a well known TLR4 ligand, was used as a positive control. As expected, under similar conditions, LPS-stimulated macrophages exhibited decreased TLR4 sialylation on the membrane in contrast to *L. donovani* infected cells. Our observations suggest some close relationship between enhanced sialylation of TLR4 and parasite infection.

Our data exhibited reduced membrane-bound Neu1 (Figure 2) and enhanced α2,3-linked sialic acids on TLR4 (Figure 3A) which intrigued us to check the effect of this enhanced sialylation on its association with Neu1 (Figure 3B). Accordingly, immunoprecipitated TLR4 was immunoblotted with anti-Neu1 antibodies. We observed Neu1-TLR4 association was reduced significantly in parasite-infected macrophages than its uninfected counterpart. In contrast, LPS-stimulated macrophages exhibited just opposite effect indicating a role of Neu1 in the enhancement of TLR4 sialylation.

So far we have observed enhanced sialylation of TLR4 possibly due to reduced Neu1-TLR4 association in parasite-infected cells (Figure 3B). This prompted us to investigate whether such hyper sialylation of TLR4 affects its downstream signaling. At the initial stage, TLR4 signaling involves its association with the key adaptor molecule MyD88 for its further activation. To address this, we checked the recruitment of TLR4 with MyD88 (Figure 3C).

Accordingly, immunoprecipitated TLR4 was immunoblotted with anti-MyD88 antibodies. We observed a reduction in TLR4–MyD88 association in infected macrophages indicating some involvement of enhanced sialylation of TLR4 during parasite infection. However, LPS-treated cells showed a better association of TLR4 with MyD88 indicating proper initiation of downstream signaling.

### Effect of Neu1 Overexpression in Parasite-Infected Macrophages

**Figure 4:**
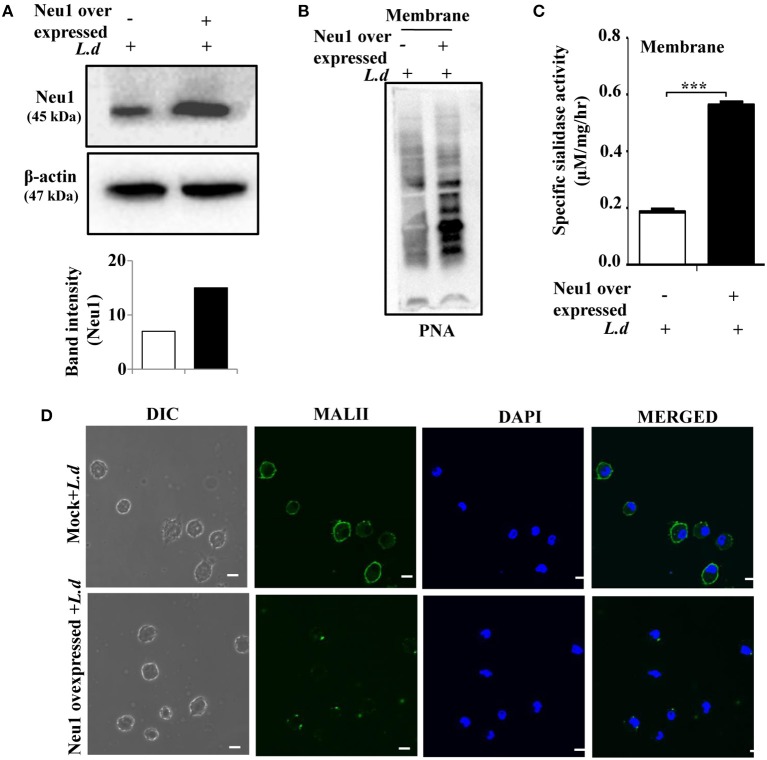
Neu1 overexpression reduces sialylation on infected macrophages. **(A,B)** J774.A1 cells were either mock-transfected or transfected with Neu1 plasmid and infected as described in Materials and Methods. Cells lysates were analyzed by western blot and probed with anti-Neu1 antibody. β-actin was used to show equal loading. The densitometric score for each band showed enhanced Neu1 expression in Neu1-transfected infected cells. Membrane fractions of these cells were analyzed similarly and the blot was probed with PNA **(B)** to further ensure higher Neu1 expression in Neu1-transfected *L. donovani* infected cells. **(C)** The enzyme activity of membrane protein of Neu1-transfected *L. donovani*-infected cells was measured as stated in Materials and Methods. Mock-transfected infected cells were used for comparison. Enhanced sialidase activity in the membrane fraction of Neu1-transfected *L. donovani*-infected cells was observed. Data were obtained from three independent experiments and expressed as mean values ± SD. Significance ***p ≤ 0.001. **(D)** Mock and Neu1-transfected infected cells were stained with FITC-MALII/DAPI and visualized the status of sialic acids on the cell membrane of these Neu1-transfected infected cells in a confocal microscope. Scale bar = 20 μM.

To specifically pinpoint the effect of Neu1 on TLR4 during parasite infection, we overexpressed Neu1 in macrophage by transfecting a plasmid encoding the Neu1 gene followed by infection with parasite (Figure 4A). Enhanced Neu1 in the Neu1-transfected cells ensured its overexpression compared to the mock-transfected cells. Penultimate sugar of terminal sialic acid moiety on sialoglycoconjugate is usually galactose. Accordingly, we checked the binding of a galactose-binding lectin (PNA) with Neu1-transfected parasite-infected cells (Figure 4B). PNA blotting depicted an enhanced binding with exposed galactose units on the Neu1-transfected cells due to the removal of sialic acids by the increased Neu1 compared to mock-transfected cells. To further confirm the overexpression, we measured the sialidase activity of the membrane fractions of Neu1-transfected cells (Figure 4C). Approximately 3.2-fold (*p* ≤ 0.001) increased enzyme activity was observed in the membrane fraction of Neu1-transfected cells compared to mock transfection during *L. donovani* infection.

Furthermore, we checked the effect of overexpressed Neu1 on the parasite-infected macrophages by confocal microscopy (Figure 4D). A decreased level of MALII-binding suggested decreased α2,3-linked sialic acids on the cell surface of Neu1-transfected parasite-infected cells. All these observations confirmed the overexpression status of Neu1 in parasite-infected cells.

### Neu1 Overexpression Removes TLR4 Sialylation Leading to Activation

**Figure 5:**
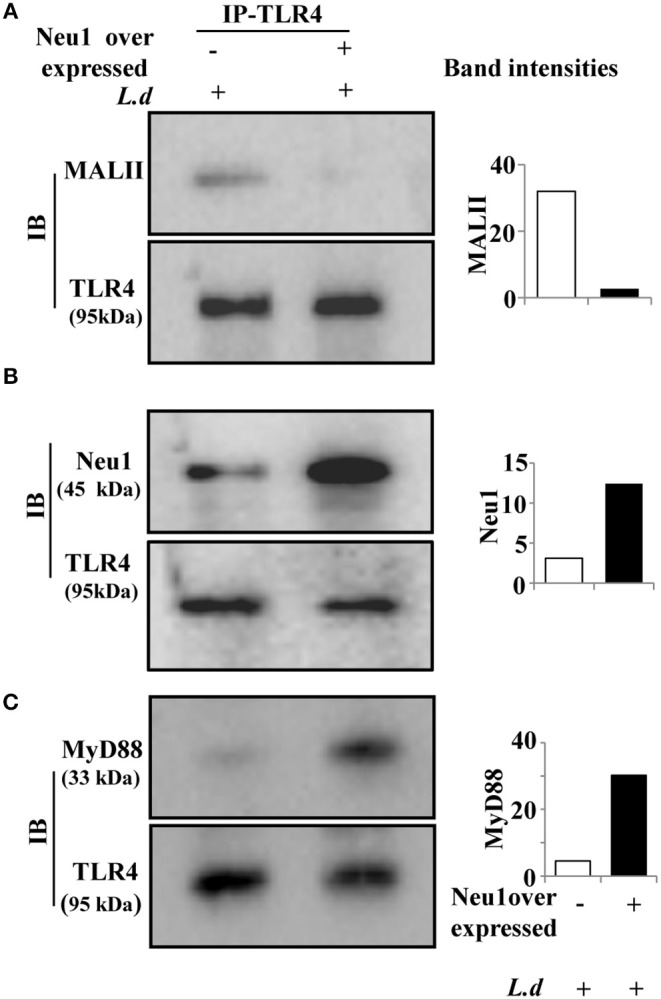
Neu1 overexpression led to TLR4 desialylation in infected macrophages. **(A–C)** Cell lysates from mock and Neu1-transfected and infected J774.A1 cells were incubated with anti-TLR4 antibody and the immune complexes were pulled down with Protein A and run on SDS-PAGE. The blots were probed with biotinylated MALII **(A)**, anti-Neu1 **(B)**, and anti-MyD88 **(C)** antibodies demonstrating reduced sialylation on TLR4 which exhibited its enhanced association with Neu1 and MyD88. Data were obtained from three independent experiments and representative blots are shown. The densitometric score for each band was shown in bar graphs.

We next checked the sialylation status of TLR4 in Neu1-transfected cells after *L. donovani* infection. Therefore, we immunoprecipitated the cell lysates from Neu1-transfected cells with anti-TLR4 antibody and immunoblotted with MALII. We observed decreased sialylation of TLR4 in the Neu1-transfected cells compared to mock transfection during infection (Figure 5A). Furthermore, we checked the association between Neu1 and TLR4 in Neu1-transfected cells in infected condition. As expected, we observed an enhanced Neu1-TLR4 association in Neu1 overexpressed condition (Figure 5B). Moreover, infection with *L. donovani* in Neu1-transfected cells showed enhanced TLR4-MyD88 association (Figure 5C). All these findings suggest that infection after Neu1 overexpression leads to TLR4 activation through its desialylation. This further confirms Neu1 does play an important role in cellular activation which is otherwise hampered during *L. donovani* infection.

### Overexpressed Neu1 Activate MAP Kinase Pathway and Enhanced Nuclear Translocation of p-65, a Functional Subunit of NFκB

**Figure 6:**
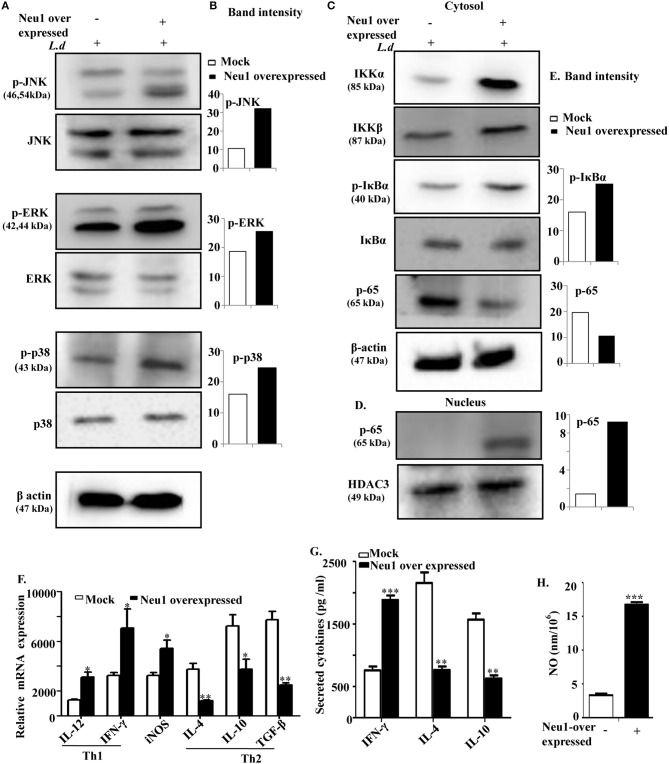
Neu1 overexpression in parasite-infected macrophages modulated MAP kinase signaling molecules and enhanced effector functions. **(A–E)** Cell lysates from Neu1-transfected and infected J774.A1 cells were processed for western blotting using specific antibodies of p-JNK, JNK, p-ERK, ERK, p-p38, p38 as stated in Materials, and Methods **(A)**. The band density was also determined **(B)**. Status of a few other signaling molecules (IKKα, IKKβ, p-IκBα, IκBα, p-65) involved in translocation of p-65 to nucleus was also determined by using respective antibodies **(C)**. Similarly, nuclear fractions were processed to determine the translocation of p-65 from cytosol to nucleus **(D)**. The band intensity of each band was plotted **(E)**. β-actin and HDAC3 were used as loading control for cytosol and nucleus, respectively. Thus, Neu1-transfected *L. donovani*-infected cells modulated MAP-kinase signaling molecules. **(F)** Relative mRNA expression of Neu1-transfected and mock-transfected J774A.1 cells was determined using specific primers for Th1 (IL-12 and IFNγ), Th2 cytokines (IL-4, IL-10, and TGF-β), and iNOS as stated in Materials and Methods. Values were normalized against 18s rRNA. **(G,H)** Cell-free culture supernatant from *L. donovani*-infected mock and Neu1-transfected cells was used to determine the secreted cytokines using respective ELISA kits **(G)** and nitric oxide level by Griess assay **(H)**. Data were obtained from three independent experiments and expressed as mean values ± SD. Significance *p ≤ 0.05, **p ≤ 0.01, and ***p ≤ 0.001.

Enhanced Neu1-TLR4 and TLR4-MyD88 associations in Neu1-overexpressed cells further intrigued us to explore the role of sialic acids on TLR4 in TLR4-mediated downstream signaling during parasite infection. We, therefore, checked the status of different signaling molecules of the MAP-kinase pathway in Neu1-transfected cells after *L. donovani* infection. We observed enhanced phosphorylation of JNK, ERK1/2, and p38 MAPK in Neu1-transfected cells compared to mock-transfected cells (Figure 6A). The band intensities of the blots are shown (Figure 6B).

Nuclear translocation of a transcription factor NFκB leads to transcription of proinflammatory genes. We, therefore, assessed the nuclear localization of the functional subunit of NF-κB namely p65 (Figures 6C,D). The relative band intensities of the blots are shown (Figure 6E). Parasite infection of Neu1-transfected macrophages increased the expression of IKKα and IKKβ in the cytosolic fraction. This enhanced IKKα and IKKβ lead to enhanced phosphorylation of IκBα, causing its degradation and ultimately sets free the functional subunit of NF-κB, p65. This lead to a reduced p-65 in the cytosol (Figure 6C) due to enhanced nuclear translocation (Figure 6D). Thus, we observed a good correlation between Neu1-mediated desialylation of TLR4 and activation of the MAPK pathway in addition to NF-κB nuclear translocation indicating higher cellular activation thereby possible inhibition of parasite infection in presence of enhanced Neu1.

### Overexpressed Neu1 Up Regulate Proinflammatory Cytokines in *L. donovani* Infected Cells

Enhanced nuclear translocation of functional transcription factor (p65 subunit), generally leads to enhanced generation of Th1 cytokines. Therefore, we have assessed both Th1 and Th2 cytokines in Neu1-transfected cells upon *L. donovani* infection (Figure 6F). We observed an enhanced genetic expression of the Th1 cytokines namely IL-12 (~2.6-fold, *p* ≤ 0.05) and IFNγ (~1.9-fold, *p* ≤ 0.05) with down-regulated expression of Th2 cytokines IL-4 (~2.1-fold, *p* ≤ 0.01), IL-10 (~1.8 fold, *p* ≤ 0.05), and TGFβ (~3.3-fold, *p* ≤ 0.01) in Neu1-transfected cells.

Additionally, we also measured the level of a few secreted cytokines in the cell supernatant of Neu1-transfected parasite-infected cells by ELISA (Figure 6G). The level of the secreted Th1 cytokines IFNγ was also significantly (*p* ≤ 0.001) increased by ~2.6-fold in Neu1-transfected parasite-infected cells. In contrast, there was a significant (*p* ≤ 0.01), reduction of the Th2 cytokines IL-10(~2.6-fold) and IL-4 (~2.8-fold, *p* ≤ 0.01) compared to infected mock-transfected cells. This observation well-corroborated with the genetic expression data.

Nitric oxide is an important effector molecule involved in host defense against the parasite. Therefore, we checked the genetic expression of *iNOS* gene. We observed a significantly (*p* ≤ 0.05) enhanced *iNOS* expression of 2.3-fold in Neu1-transfected cells after parasite infection (Figure 6F). Additionally, secreted nitric oxide (NO) level in the supernatant was determined by Griess assay (Figure 6H). Approximately, ~4-fold enhancements in the NO level (*p* ≤ 0.001) were found in parasite-infected Neu1-transfected cells compared to its mock-transfected counterpart.

### Neu1 Overexpression Reduces Parasite Burden in Infected Cells

So far we have demonstrated that Neu1-transfection induced enhanced TLR4-mediated activation of several signaling molecules in the MAPK pathway which leads to enhanced Th1 cytokines along with *iNOS* and NO suggesting increased effector immune response in these parasite-infected cells. Therefore, the next obvious step was to check the actual status of the parasite burden in these Neu1-transfected cells.

**Figure 7:**
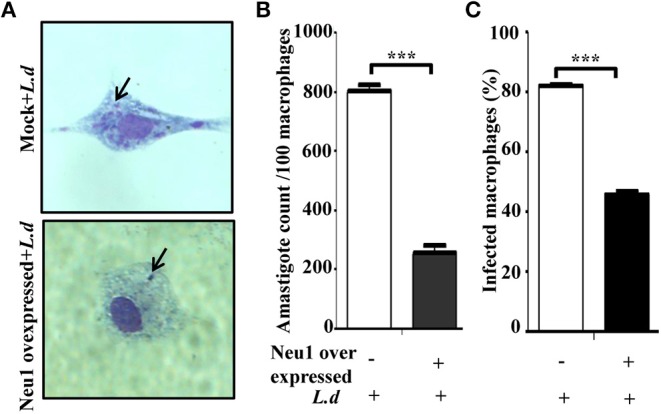
Effect of Neu1 transfection on survival of *Leishmania donovani* inside macrophages. **(A–C)** Parasite burden inside macrophages was determined in the peritoneal macrophages from BALB/c mice. Cells were infected and processed for Giemsa staining as described in Materials and Methods. Intracellular amastigotes were visualized by optical microscopy to determine parasite load **(A)**. The purple dots indicated by black arrow demonstrated amastigotes in Neu1-transfected cells. The parasite load inside cells was measured by counting the number of intracellular amastigotes per 100 macrophages **(B)**. The rate of infection was also analyzed by counting the percent infected macrophages **(C)**. Each determination was made in triplicate and the values were expressed as mean ± SD for three independent experiments. A Student's *t*-test was used to evaluate statistical significance; ***p ≤ 0.001.

Accordingly, resident peritoneal macrophages cells were transfected with Neu1 plasmid followed by *L. donovani* infection and the parasite load was determined (Figure 7A). We observed a decreased number of intracellular amastigote in the Neu1-transfected cells after staining with Giemsa (purple dots indicated by black arrow) than mock-transfection. Corroborating with this data, there was also a significant (*p* ≤ 0.001) reduction in the number of amastigotes per 100 macrophages in Neu1-transfected cells (Figure 7B). This was further reflected by a significant (*p* ≤ 0.001) reduction in the percentage of infected macrophages in the Neu1-transfected cells compared to mock counterpart suggesting effective activation of the immune response leading to parasite killing due to overexpression of Neu1 (Figure 7C).

### Neu1 Silencing Reduced TLR4-Neu1 Association

**Figure 8:**
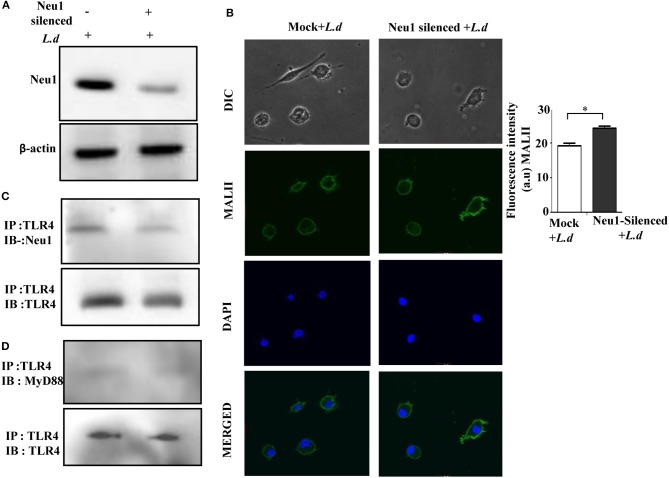
Neu1-silencing reduced TLR4-Neu1 association. **(A)** Cells lysates from mock and Neu1-silenced parasite-infected cells were processed for western blot using anti-Neu1 antibodies **(A)**. Additionally, the cell lysate was also incubated with anti-TLR4 antibody and immunoprecipitated with protein A beads. The precipitates were analyzed by SDS-PAGE under non-reducing conditions and subsequently probed with anti-Neu1 **(C)** and anti-MyD88 **(D)** antibodies. **(B)**. Mock and Neu1-silenced infected cells were stained with FITC-MALII and DAPI and visualized in an inverted confocal microscope to determine the status of α2,3-linked sialic acids on the cell membrane. Scale bar = 20 μM. Fluorescence intensity graph is shown. Each determination was made in triplicate and the values were expressed as mean ± SD for three independent experiments. A Student's *t*-test was used to evaluate statistical significance; *p ≤ 0.05.

So far we have demonstrated that Neu1-overexpression resulted in activation of the innate immune response in the parasite-infected cells. This led us to further investigate the effect of Neu1 silencing on TLR4 during this parasite infection. Accordingly, the cells were transfected with Neu1 shRNA and thereafter infected with the parasites. We observed reduced Neu1 protein level expression in these transfected cells compared to the mock thus confirming Neu1 silencing (Figure 8A). The Neu1-silenced parasite-infected cells exhibited an enhanced MALII binding (*p* ≤ 0.05) when compared to the mock-transfected infected macrophage by confocal microscopy (Figure 8B). The fluorescence intensity graph is shown.

As expected, we observed a reduced Neu1-TLR4 association in the Neu1-silenced parasite-infected macrophages compared to mock-infected cells (Figure 8C). Earlier, we have observed decreased TLR4-MyD88 association in infected cells (Figure 3C), here we were unable to observe any further detectable reduction in association of TLR4 with MyD88 after Neu1 silencing (Figure 8D). This observation indicates reduced Neu1 on the parasite-infected cells is indeed responsible for impaired TLR4 activation.

### Neu1-Cathepsin a Association Is Modulated During Infection

**Figure 9:**
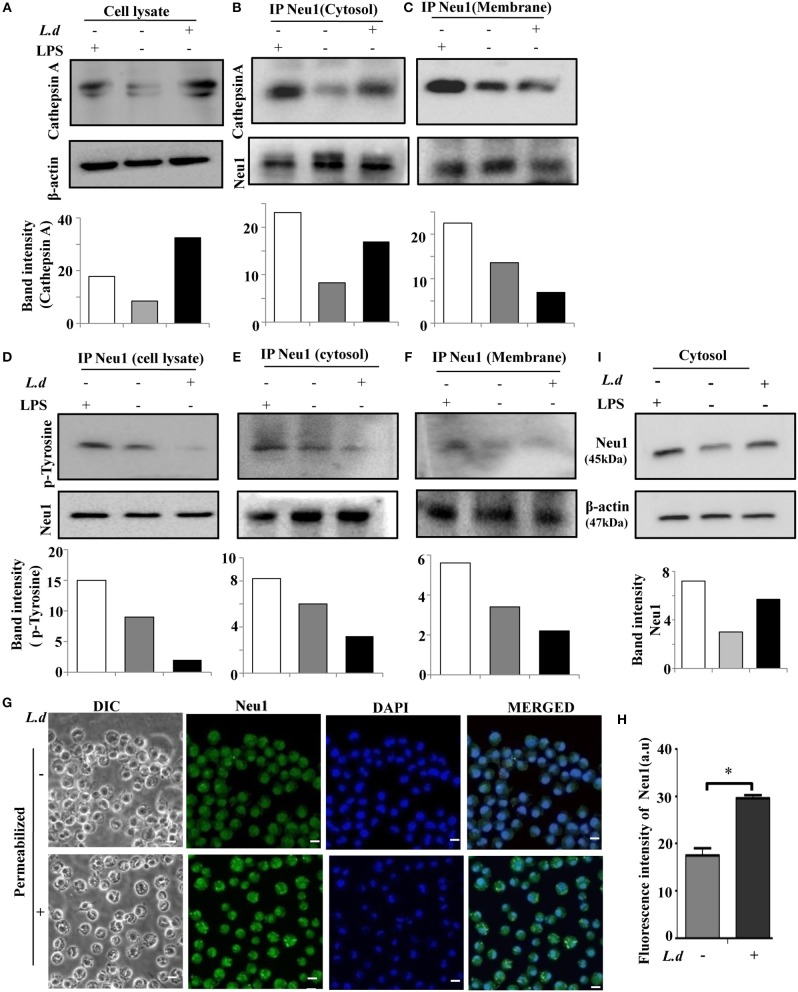
Reduced Neu1-cathepsin A association and tyrosine phosphorylation of Neu1 on membrane of *L. donovani* infected cells. **(A)** Lysates from uninfected or infected cells were processed for western blotting and the blot was probed with anti-cathepsin A antibody to determine the level of cathepsin A during infection. Representative blot exhibited enhanced cathepsin A during *L. donovani* infection. **(B,C)** Membrane and cytosolic fractions of these cell lysates, as well as the total lysate **(D)**, were incubated with anti-Neu1 antibody overnight and immunoprecipitated. The immune complex was run on SDS-PAGE and subsequently blotted with anti-cathepsin A and anti-p-Tyrosine antibodies both for the cytosolic **(B,E)** and membrane **(C,F)** fractions, respectively. Additionally immunoprecipitates from total cell lysate was blotted with anti-p-tyrosine antibody **(D)**. Reduced Neu1-cathepsin A association in membrane compared to cytosol along with overall reduction of Neu1-p-Tyrosine association was depicted in infected cells. **(G,H)** Adhered cells were infected and processed for microscopy. These were fixed, permeabilized, and stained with anti-Neu1 antibody, followed by Alexafluor 488 secondary antibody and was visualized in a Zeiss inverted confocal microscope **(G)**. The fluorescence intensity of cytosolic Neu1 was determined **(H)**. Scale bar = 20 μM. Representative slides demonstrated enhanced cytosolic Neu1 during *L. donovani* infection. **(I)** Status of Neu1 protein in cytosolic fractions of uninfected and infected cells was determined by western blot analysis using anti-Neu1 antibodies. β-actin was used as loading control. Additionally, LPS-stimulated macrophages were also used as an internal control. Enhanced Neu1 was found in the cytosol of the infected cells. Data were derived from three independent experiments and presented as mean values ± SD. Significance *p ≤ 0.05.

Neu1 is usually complexed with β-galactosidase and the serine carboxypeptidase protective protein/cathepsin A. Cathepsin A is essential for the catalytic activity of Neu1. So far we have observed a decrease in Neu1 activity in the membrane fraction of infected macrophages with hypersialylation in TLR4. Therefore, we checked the status of cathepsin A in *L. donovani* infected macrophages. An enhanced level of cathepsin A was demonstrated during this infection (Figure 9A). Furthermore, immunoblotting of cytosolic Neu1 with cathepsin A exhibited an increased association in the cytosolic fraction (Figure 9B) which was reversed when membrane fraction was used (Figure 9C) suggesting less available Neu1-cathepsin A association on the membrane of *L. donovani* infected macrophages.

### Impaired Phosphorylation on Neu1 Prevent Its Cell Surface Translocation

It has been reported that in activated lymphocytes Neu1 phosphorylation leads to its membrane translocation. To address if such phosphorylation plays any role in surface translocation of Neu1 during infection, we immunoprecipitated Neu1 from the cell lysate of uninfected and infected cells with anti-Neu1 antibody and immunoblotted with anti-phosphotyrosine antibody. We observed a decreased level of Neu1-phosphorylation in cell lysate during infection (Figure 9D). A similar trend was observed both in the cytosol (Figure 9E) and membrane (Figure 9F) fractions. This was corroborated by the fluorescence microscopy (Figure 9G). The mean intensity graph of intracellular Neu1 is also shown (Figure 9H). Western blot analysis further revealed more cytosolic Neu1 during infection (Figure 9I). Thus, this reduced tyrosine phosphorylation may probably be one of the reasons for preventing the translocation of Neu1 to the cell surface of *L. donovani* infected macrophages.

## Discussion

The surface of immune cells is densely ornamented with sialic acids. Alteration in cell surface sialic acids causes marked changes in their behavior by modulating the interaction with pathogens and the activity of immune cells (5, 56). TLR4, an important molecule of the innate immune arm on the host cell surface is a highly sialylated molecule predominantly decorated with α2,3-linked sialic acids. However, the correlation of sialylation on TLR4 and establishment of successful *L. donovani*. infection in macrophages was not adequately studied. Here, we have established a specific novel role of a mammalian lysosomal sialidase (Neu1) on the regulation of TLR4 sialylation and its impaired functioning during this parasite infection.

The major achievement of our study is to provide evidence that sialidase Neu1 with substrate specificity toward α2,3-linked sialic acids, is reduced on the membrane of *L. donovani* infected macrophages leading to decreased TLR4-Neu1 association. Reduced tyrosine-phosphorylation of Neu1, as well as decreased availability of Neu1-cathepsin A complex possibly plays a significant role in its reduced translocation from cytosol to membrane. As a result, it failed to cleave the α2,3-linked sialic acids on TLR4 resulting in reduced TLR4-MyD88 complex formation which was unable to activate subsequent downstream signaling. However, Neu1 overexpression reverses this effect. In this condition, TLR4 effectively could activate downstream MAP-kinase signaling pathway with enhanced nuclear translocation of NFκB that resulted in increased Th1 cytokines and nitric oxide secretion which ultimately leads to reduced parasite burden in infected macrophages. Furthermore, parasite-infection after Neu1 silencing resulted in a reverse scenario. Therefore, decreased Neu1 and increased α2,3-linked sialic acids on TLR4 plays an important role to ascertain successful parasite infection in the host cells. Taken together, our study, for the first time, establishes a link between impaired translocation, reduced Neu1 and perturbation of sialylation on TLR4 thus demonstrating an inverse correlation between hypersialylation and impairment of TLR4 signaling during *L. donovani* infection.

Our initial study revealed upregulated α2,6 and α2,3-linked sialic acids on the cell surface of *L. donovani* infected macrophages. This was further corroborated by decreased expression and enzyme activity of Neu1 on the membrane of *L. donovani*. infected cells. Interestingly, one of the upregulated α2,3-linked sialoglycoprotein on the surface of *L. donovani*. infected macrophages exhibited an apparent molecular weight of ~100 kDa which matched with a membrane receptor TLR4. This hinted us a possible correlation of enhanced sialylation of TLR4 on macrophages and *L. donovani* infection.

We had earlier established that *L. donovani* does not have any sialic acid synthesis mechanism of its own (17, 46). They adsorb sialic acids from the host itself. Subsequent report indicated two putative Leishmnaial genes that may be involved in sialic acid biosynthesis in the parasite (16). We have recently reported that parasite use these acquired sialic acids to enter into the macrophage through sialic acid binding immunoglobulin like lectins commonly named as siglecs (41). However, under the experimental condition, we have excluded the possibility of the presence of leishmanial sialic acids bound on the macrophage membrane. Moreover, comparing the 10-fold larger size of the macrophages (~20 μm) to that of the parasite (2 μm), contribution of sialic acids (if any) from the promastigotes is negligible. Furthermore, we have validated that the enhanced sialylation observed was reflected on TLR4 which is specifically present on the host membrane. There is no report so far indicating direct binding of the *L. donovani*. parasite with TLR4. This apparently rules out the contribution of parasite sialic acids in the association of sialylated TLR4 and MALII. Therefore, the presence of enhanced alpha 2,3-linked sialic acids on the membrane of infected macrophage as well as sialylated TLR4 are mainly due to the modulation of sialidases in the host and not possibly from the parasite.

Szewczuk et al. demonstrated decreased sialylation of TLR4 on LPS-treated macrophages which is due to enhanced Neu1 causing the removal of α2,3-linked sialyl residues, a prerequisite to removing steric hindrance leading to receptor association (33). Subsequently, they showed that the sialidase activity of Neu1 is an important link in the initial activation of TLR4 (40). Inhibition of Neu1 by a sialidase inhibitor, Tamiflu, reverses this effect. Similarly, Cross et al. have also reported that removal of sialyl residues from TLR4 monomers enhanced its dimerization and association with the adaptor MyD88 essential for signal activation and subsequent NFκB activation in LPS-treated macrophages (57).

Both Neu1 and TLR4 reside on the cell surface of immune cells. Therefore, an association of the two must be an essential requirement for its desialylation leading to the activation of this receptor. Here, we observed *L. donovani* infection manipulate this association by inhibiting TLR4 desialylation. Thus, we have established a possible link between the enhanced TLR4 sialylation mediated by reduced membrane-bound Neu1 during this parasite infection.

Mobilization of Neu1 from the lysosomes to the membrane is an essential criterion for proper activation of several cell surface receptors for modulating sialyl residues. Indeed, Neu1 is translocated from lysosome to the cell surface in con A-activated PBMC (58), anti-CD3 or anti-CD28 activated T-lymphocytes where this enzyme is mostly associated for the hyposialylation of glycoconjugates and production of IFNγ (34). Additionally, phorbol 12-myristate 13-acetate (PMA) is also able to translocate Neu1 on neutrophil which plays an important role in the adhesion process (59, 60). Similar event takes place during differentiation of monocyte to macrophages, in which translocated Neu1 participates in antigen presentation and also influence intercellular interactions with enhanced capacity to engulf bacteria by increasing cytokine production after PMA stimulation (32). Furthermore, Neu1 is also translocated on the cell surface of LPS stimulated macrophages and dendritic cells where it is associated with TLR4 and helps to activate innate immune response (40, 61). Moreover, a missense mutation in the NEU1 gene of SM/J or SM/B10 mice strains exhibited impaired macrophage activation with reduced response (32). Therefore, it may be noted that the sialidase activity of Neu1, in general, increases significantly during cellular activation in the majority of immune cells.

However, the scenario is completely different during *L. donovani* infection. In this context, we have observed decreased Neu1-cathepsin A complex on the surface of *L. donovani* infected macrophages. More importantly, we demonstrated reduced tyrosine-phosphorylation of Neu1 on the membrane fraction of these infected cells. Therefore, we are proposing these two events play a significant decisive role in such impaired translocation solely during *L. donovani* infection. This establishes a probable correlation between the ability of Neu1 translocation on the cell surface with successful parasite infection. Such mobilization plays a significant important role in maintaining a critical level of this enzyme on the cell surface.

It is well-established that the signaling cascade initiated by the innate immune arm involving TLR4 is suppressed during *L. donovani*. infection. Here the existing lower amount of Neu1 with less enzyme activity considerably weakened its association with TLR4, demonstrating ineffective hydrolysis of α2,3-linked sialic acids on TLR4. This diminished its ability to dimerize, associate with MyD88 and therefore, impaired downstream signaling through the insufficient translocation of p65 to the nucleus with the presence of enhanced NFκB in the cytosol of *L. donovani*-infected macrophages. Thus, Neu1-TLR4 interaction is an important determinant in the outcome of TLR4 activation. Our data convincingly demonstrated a completely reversed Neu1-TLR4 association during *L. donovani* infection compared to LPS-stimulated macrophages. Thus, these parasites are playing an opposing role compared to LPS. Therefore, it seems *L. donovani*. by some unexplored mechanism, critically regulate the amount of active Neu1 available on the cell surface which is different in case of LPS/PMA-stimulation.

Besides TLR4, translocated Neu1 also plays a significant biological role by activating other cell surface receptors. Epidermal growth factor receptor (EGFR), insulin and nerve growth factor receptors are under the same regulatory control of cell surface Neu1 (62, 63). Removal of sialic acids by Neu1 leads to dimerization of these receptors and trigger subsequent signaling activation through Ras/Raf/MAP kinase pathway leading to neurite outgrowth and cell survival in neuronal cells. On the other hand, removal of sialic acids from hyaluronan receptor CD44 by Neu1 leads to enhanced Th2 cytokines in acute asthma murine model, thereby demonstrating negative regulation of the function of these sialic acids residues (64). Thus, negatively charged bulky sialic acids play context-specific critical roles in activation of the different cell surface receptors (65).

Szewczuk et al. demonstrated that Neu1 and matrix metalloproteinase-9 cross-talk is essential for TLR4 activation possibly by G protein-coupled receptor (66). However, the key player involved in TLR4 inhibition by Neu1 during this parasite-infection is largely unknown. Unlike the other sialidases, the activity of Neu1 is dependent upon its association with cathepsin A. Neu1 forms a multienzyme complex with cathepsin A and β-galactosidase (67). Cathepsin A activates Neu1 and protects them against proteolytic degradation in the lysosomes. We demonstrated a defect in this association which may be responsible for a decreased translocation of Neu1 and reduced sialidase activity on the membrane. However, such an association was slightly more in the cytosol suggesting some other possible mechanism of Neu1 translocation in the infected macrophages.

Post-translational modifications like glycosylation and phosphorylation may additionally influence the activation of Neu1 and its translocation (58). It has been reported that enhanced Neu1 phosphorylation at tyrosine moiety leads to its membrane translocation in activated lymphocytes. However, this is just reversed in case of *L. donovani* infection suggesting a defective cell surface translocation. Although exact mechanism, so far has not been identified, the role of reduced tyrosine-phosphorylation of Neu1 is possibly an important event in this translocation.

Thus, we propose an inverse correlation of decreased Neu1 on the surface of these parasite-infected macrophages and enhanced sialic acids on TLR4 with reduced Neu1-TLR4 association and reduced activation, which is a prerequisite event for establishing a successful infection. All these events lead to the impaired innate immune response along with a higher number of amastigotes in infected macrophages suggesting a context-specific role of Neu1 during *L. donovani* infection.

Moreover, in order to pinpoint this important regulation by Neu1 during infection, we over expressed Neu1. As expected, we observed reduced sialylation on TLR4 in Neu1-transfected *L. donovani* infected macrophages due to an enhanced Neu1-TLR4 association leading to enhanced TLR4 association with the signal transducer protein MyD88 and subsequent activation of the MAP kinases signaling pathway. Subsequently, we observed enhanced phosphorylation of JNK, ERK and p38 along with enhanced nuclear translocation of a functional subunit of a transcription factor, NFκB in Neu1-transfected cells after infection. Reports suggest desialylation of immune cell surface leads to its activation (7). Our data very well-corroborated with such report.

*Leishmania donovani* infection is usually dominated by Th2 immune response leading to parasite survival and persistence inside macrophages (68). As a result, proinflammatory cytokines, the signature of Th1 immune response, remains subdued that makes macrophage defense submissive (69–72). Our data revealed a strong upregulation of IL-12 and IFNγ with simultaneous down-regulation of IL-4, IL-10, and TGF-β indicating a condition favorable for promoting macrophage defense. An effective antileishmanial response was generated through a dominant Th1 cytokine response. This was reflected by an almost complete reduction of parasite burden along with enhanced NO and iNOS in infected macrophages after Neu1 overexpression. The important role of Neu1 in immune activation by desialylation was also supported by Neu1 silencing.

**Figure 10:**
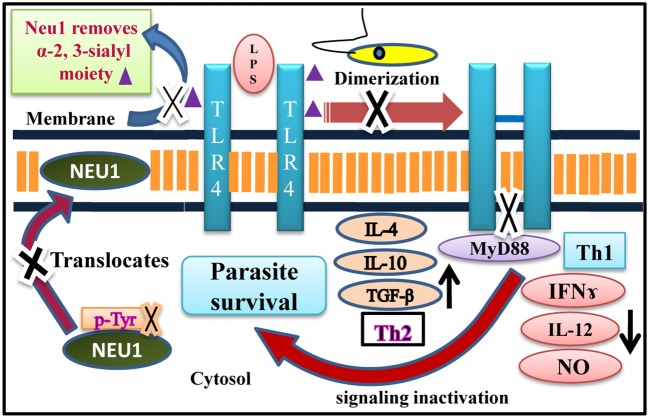
Neu1 mediated TLR4 receptor modulation during *L. donovani* infection. Schematic representation demonstrating perturbation of dimerization of two monomers of TLR4 due to the enhanced presence of bulky α-2,3-sialyl residues during *L. donovani* infection mediated by defective translocation of Neu1 from cytosol to membrane through impaired tyrosine-phosphorylation of this enzyme. Reduced level of Neu1 on *L. donovani* infected cell surface prevents its association with TLR4 responsible for its enhanced sialylation which subsequently decreased TLR4-MyD88 complex formation leading to inactivation of MAP-kinase signaling molecules which promotes enhanced Th2 and reduced Th1 cytokines and decreased iNOS/NO, a perfect condition for the survival of parasite inside the macrophages. Thus, it is a unique mechanism modulated by a sialidase (Neu1) during *L. donovani* infection as opposed to LPS-stimulated macrophages.

TLR4 is an important molecule of the innate immune response on the host membrane that initially tries to prevent the entry of pathogens by generating a robust anti-inflammatory immune response and thereby prevent successful establishment of infection. Accordingly, our main objective was to explore how and why TLR4 functioning is hampered during initial phase of infection with promastigotes which are the infective form of the parasite and encounters the host defense mechanism. Therefore, here we have only highlighted the early event of the host-parasite interaction in the context of sialylated TLR4 and sialic acid modulating enzyme (sialidase). Taken together our study ascertains the important role of enhanced cell surface Neu1 in TLR4 activation which has the potential to clear the parasite burden by generating a robust Th1 proinflammatory defense in the host cells which is otherwise reversed during infection (Figure 10). Neu1 deficiency in humans leads to the reduced ability of immune cells to produce cytokines leading to partial immunodeficiency (32). Thus, Neu1 on the cell surface may be one of the crucial factors in the suppression of the immune response during active visceral leishmaniasis in humans. This understanding of host-pathogen interaction will open up new windows in disease progression not only in *L. donovani* but for other pathogens as well. Therefore, from a therapeutic point of view, Neu1 modulation on the host cell surface may form a rationale for effective drug designing against this deadly neglected tropical disease.

## Ethics Statement

All the animal experiments were carried out in accordance with the National Regulatory Guidelines issued by Committee for the Purpose of Control and Supervision of Experiments on Animals (CPCSEA), Ministry of Environment and Forest, Government of India. Use of Syrian Golden hamsters and Balb/c mice were approved by the Institutional Animal Ethics Committee of CSIR-Indian Institute of Chemical Biology, Kolkata, India with license number 147/1999/CPCSEA. Animals were housed under standard condition such as temperature (25 ± 1°C), relative humidity (55 ± 10%) and 12 h/12 h light/dark cycles and fed with the standard diet.

## Author Contributions

JK performed all the experiments. SR conceived the work and initiated the project. CM supervised the whole work. All authors have contributed valuable comments and scientific inputs in writing the manuscript.

### Conflict of Interest

The authors declare that the research was conducted in the absence of any commercial or financial relationships that could be construed as a potential conflict of interest.
